# Expansion of non‐native plant *Flaveria bidentis* (L.) Kuntze driven by a range of factors leading to patchy distribution patterns

**DOI:** 10.1002/ece3.9303

**Published:** 2022-09-20

**Authors:** Qianmei Wu, Chengdong Xu, Jiamei Li, Wanxue Liu, Fanghao Wan, Jianying Guo, Rui Wang

**Affiliations:** ^1^ State Key Laboratory for Biology of Plant Diseases and Insect Pests Institute of Plant Protection, Chinese Academy of Agricultural Sciences Beijing China; ^2^ State Key Laboratory of Resources and Environmental Information System Institute of Geographic Sciences and Natural Resources Research, Chinese Academy of Sciences Beijing China; ^3^ College of Life Science Henan Agricultural University Zhengzhou China

**Keywords:** distribution patterns, *Flaveria bidentis* (L.) Kuntze, non‐native species, population management, range expansion

## Abstract

Given the growing concern over the ecological impacts of non‐native species, exploring these species' expansion edge and distribution patterns and their driving factors is important for developing suitable management measures. *Flaveria bidentis* (L.) Kuntze, a non‐native plant that was introduced to China in the 1990s, has spread from southern Hebei Province, where it first took root, to the surrounding regions and has become one of the most notorious invasive weeds in northern China. Based on 15 years (2006–2021) of extensive field investigations, the spatial distribution of sampling and occurrence points were mapped in the recently expanded region of *F. bidentis*' population. Then, nearest neighbor analysis is used to characterize the spatial pattern differences between samplings and occurrences. An exponential decay function was used to elucidate the driving factors contributing to the presence and absence of *F. bidentis*. Our results demonstrated an effective random sampling setup, a heterogeneous spatial distribution of *F. bidentis*, and a multi‐regional independent aggregation distribution pattern (*p* < .01). There were significant spatial correlations between the aggregation areas of plant occurrence points and the locations of roads and construction sand distribution centers. These findings suggest that human activities involving major roads and construction sand distribution centers were driving factors contributing to this long‐distance dispersal and spatially discontinuous distribution patterns. The presence of these patchy distribution patterns has important implications for ongoing efforts to manage populations of non‐native species.

## Graphical abstract

Understanding the spatial distribution patterns and its driving factors in newly invaded areas is the key to early management of invasive alien species. Here, we explored the distribution patterns of invasive alien plant Flaveria bidentis (L.) Kuntze in its expansion frontier in northern China based on extensive field samplings. Our results demonstrated a heterogeneous spatial distribution of F. bidentis, and a multi‐regional independent aggregation distribution pattern. There were significant spatial correlations between the aggregation areas of plant occurrence points and locations of roads and construction sand distribution centers. These findings suggest that human activities involving major roads and construction sand distribution centers were driving factors contributing to the long‐distance dispersal and spatially discontinuous distribution patterns.

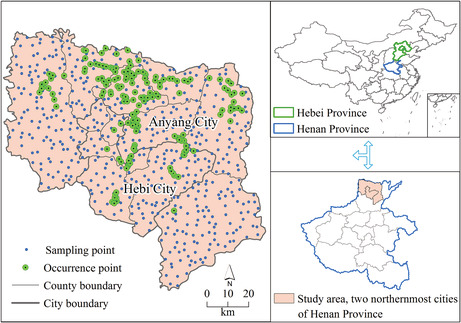

## 1 INTRODUCTION

With the rapid growth in global trade, opportunities for non‐native species to be introduced to new habitats are increasing (Anderson et al., 2015; Early et al., 2016). Once a recently introduced species has successfully been established in a new area, it will continue to occupy the new habitat and the species range will increase to its ecological limits. When non‐native species become widespread, they not only lead to a reduction and loss of biodiversity but can also threaten local ecological and economic development (Curnutt, 2000; Petsch, 2016; Pimentel et al., 2000). Therefore, one of the most effective management strategies is to eradicate the non‐native population on the edge of the population range (Choi et al., 2017; Natale et al., 2012; Sharov & Liebhold, 1998).

The primary challenge of this eradication approach is to effectively locate newly established populations on the edge of the range. In order to do so, it is imperative to understand a plant's dispersal and growth patterns at a population level. Generally, the distribution patterns of younger populations where the range of a non‐native plant has expanded into a new habitat are typically either continuous or discontinuous (Mistro et al., 2012; Petrovskaya et al., 2017; Petrovskii et al., 2005; Rodrigues et al., 2015). The seminal research results obtained by Kolmogorov et al. (1937) and Skellam (1951) demonstrate the existence of a continuously spreading front for non‐native species, which separates already existing populations and areas where non‐native species do not exist. However, in recent decades, an alternative model of spatial distribution patterns has been described and observed in which the spreading edge is discontinuous and patchy; some isolated patches have strong populations of non‐native species, whereas others nearby are sparsely populated or not populated at all (Rodrigues et al., 2015). Recognizing different spatial distribution patterns is critical for controlling the range expansion of non‐native species (Petrovskaya & Zhang, 2020a, 2020b). The non‐native species that are continuously distributed can be detected and eradicated, beginning along front spatial distribution and opposite their expanding range direction. In contrast, non‐natives who are discontinuously distributed require eradication efforts to identify areas with separated disparate dense patches. However, discontinuously distributed species are spatially heterogeneous, which requires more careful resource allocation than continuous front spatial distributions (Petrovskaya & Zhang, 2020a, 2020b). Especially in the 21st century, human activities have facilitated the long‐distance dispersal of non‐native species, making their distribution patterns both more complex and discrete, which significantly increases the challenges related to controlling non‐native plant populations (Bullock et al., 2018; Capinha et al., 2015; Horvitz et al., 2017; Rew et al., 2018; Robinet et al., 2017; Von der Lippe et al., 2013). Therefore, accurate identification of spatial patterns, especially discontinuous distribution, could improve eradication efforts by enabling targeted control measures that can prevent a non‐native species from further spreading after successful persistence in an area and allocated management efforts to protect ecosystems that do not yet contain the non‐native species (Lewis et al., 2016; Lodge et al., 2006).

Therefore, comprehensive knowledge of the species distribution patterns of recently populated areas is of both theoretical and practical importance. However, several challenges remain due to methodological and data limitations (Alonso, 2019; Jarrad et al., 2015; Rosenberg & Anderson, 2016; Zhao et al., 2019). For example, simple visual observation may result in misidentification of distribution patterns; an agglomeration of isolated patches situated close to each other may appear to be continuous front spatial distribution, even though it is actually a patchy spatial pattern (Petrovskaya & Zhang, 2020a, 2020b). Many theoretical research efforts focus on the classification of spatial patterns by modeling the spatio‐temporal dynamics of non‐native species (Lewis et al., 2016; Morozov et al., 2006; Petrovskii et al., 2005; Rodrigues et al., 2015). For example, mathematic models such as the reaction–diffusion model (Petrovskii et al., 2005) and the integro‐difference mathematical model (Petrovskaya et al., 2017) have been used to simulate the spatio‐temporal dynamics of populations of non‐native species and generate a variety of visually similar spatial; however, some were continuous front distributions and others were patchy spatial patterns. Based on such simulations, several topological characteristics of spatial patterns, including the number of patches, the fragmentation rate, and the density of patches, were investigated to evaluate which ones can be used to distinguish between continuous and discontinuous spatial distributions. Researchers found that of the several topological characteristics of spatial patterns to describe various spatial density distributions, the number of patches is the most reliable factor in predicting distribution type (Petrovskaya & Zhang,  2020b). In this model, patches were defined by a region of non‐zero density with a closed external boundary (front). A continuous front density distribution can be classified as a single patch, while a no‐front patch invasion presents a collection of separate patches in one spatial domain. However, in the real world, detailed population density data are rarely available for most species due to both time and economic limitations (Tobin et al., 2007). Researchers usually only have data on the occurrence location of species, particularly in the recently populated front and edge areas. Spatial analysis methods may be used to analyze density patterns generated by a points pattern process (Fortin & Dale, 2005).

In ecological studies, nearest neighbor analyses (NNA) have been used to evaluate the distribution patterns of point data by calculating the distance between the nearest points and circling the points as isolated patches if the distances between them are significantly smaller than the mean distance computed, under the assumption that the points are distributed randomly (Clark & Evans, 1954). Furthermore, this approach employs a Monte Carlo simulation to test the statistical significance. Thus, this method is both reliable and convenient; however, it assumes that available information about the spatial distribution is sufficient to accurately reconstruct a visual image of spatial patterns. Therefore, this implies that the number of sampling points must be dense enough to capture sufficient information about the population distribution adequately and accurately represent the spatial distribution pattern (Petrovskaya & Zhang, 2020a, 2020b). However, field survey data often fails to meet these aforementioned requirements because financial and labor constraints can prevent biologists from sufficient sampling. The amount of information about the population density depends on the number of sampling locations used in monitoring.

**FIGURE 1:**
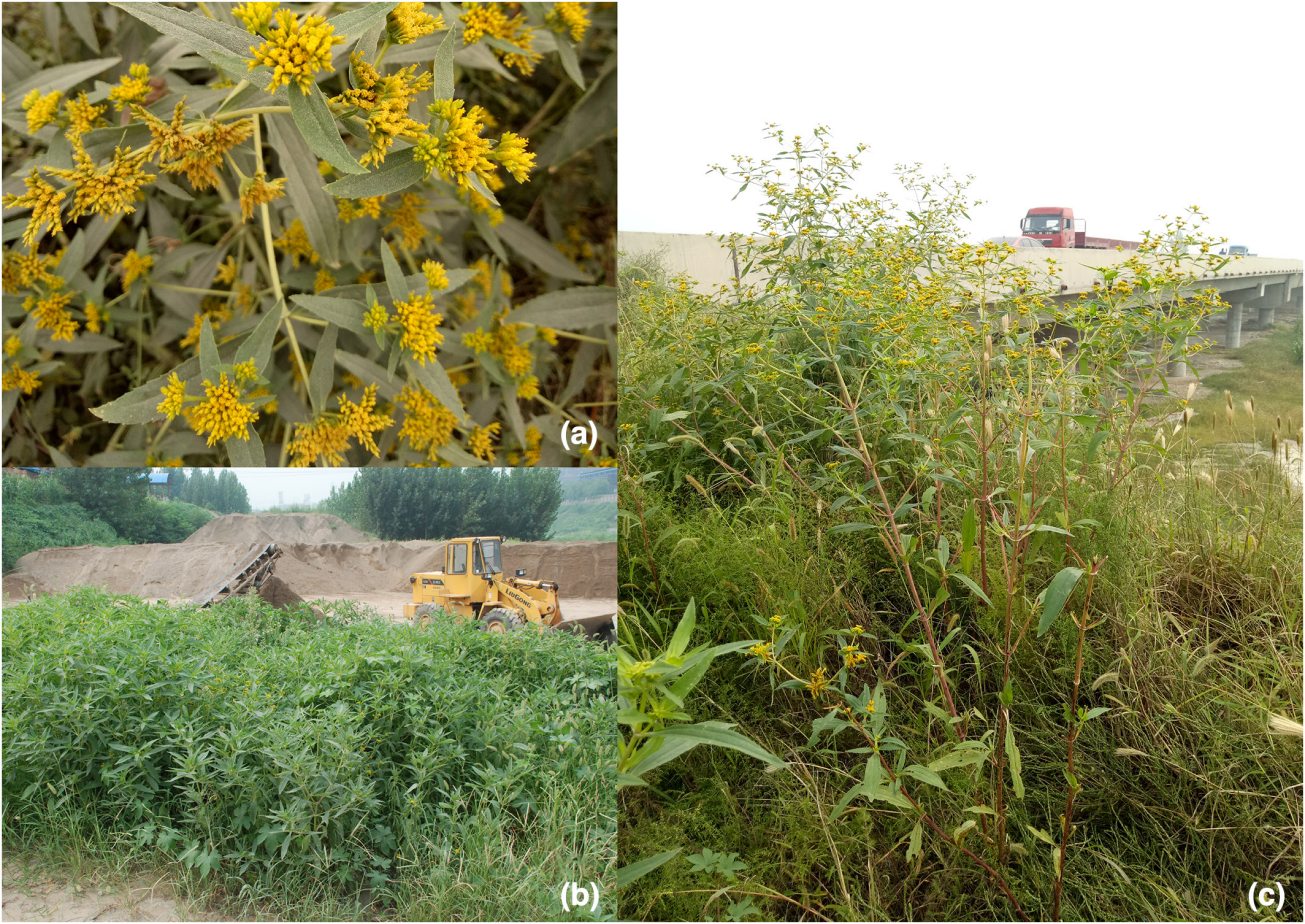
*Flaveria bidentis* (L.) Kuntze is an annual invasive plant with lanceolate‐elliptic leaves and yellow capitula (a). *F. bidentis* in the invaded area was mainly distributed in habitats associated with human activities, such as construction sand distribution centers (b) and roadsides (c).

**FIGURE 2:**
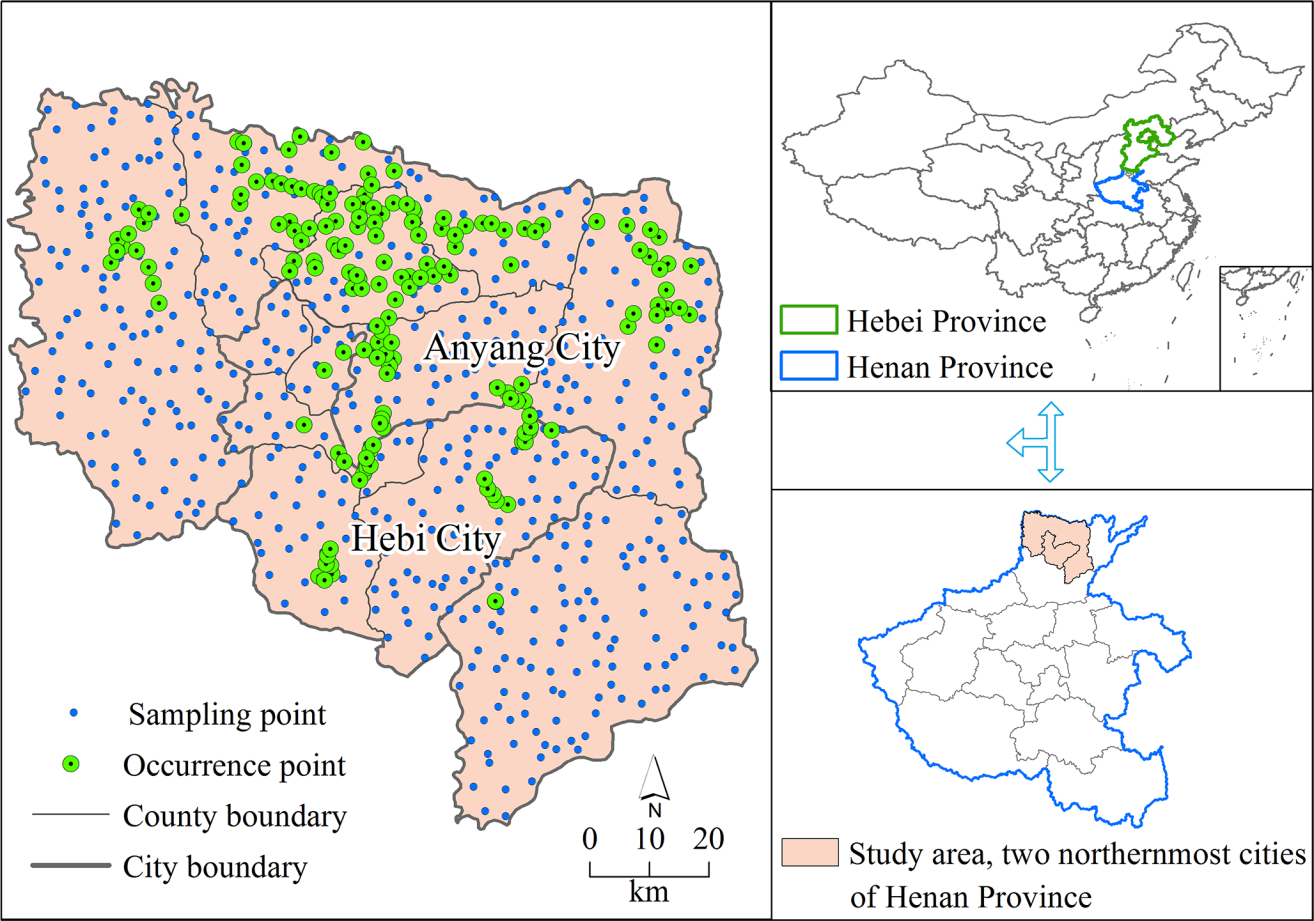
Spatial distribution of sampling and occurrence points of *Flaveria bidentis* in the study area.

The ecological challenges caused by non‐native species are of great concern to local governments, agricultural departments, and academic societies, and a large quantity of detailed data are available from government documents about populations of these non‐native species from routine monitoring and scientific field investigations. For example, *Flaveria bidentis* (L.) Kuntze is a plant native to South America that was introduced to North China in the 1990s (Figure 1). Because of its strong reproductive and growth capabilities, once it is introduced to a region, it usually forms a monodominant community and harms agricultural production and biodiversity conservation (Fan et al., 2018; Huangfu et al., 2015; Qi et al., 2019; Wang, Xian, et al., 2011; Yan et al., 2016; Zhang et al., 2012; Zhen et al., 2016). In recent years, *F. bidentis* has spread from where it was introduced in southern Hebei Province, North China, to the surrounding regions, becoming one of the most notorious non‐native weeds in northern China (Gao et al., 2004; Zhang et al., 2017; Zheng et al., 2018). Henan Province borders Hebei Province to the north; thus, northern Henan Province contains the southward spreading edge of the *F. bidentis* population (Figure 2). Since its entry into the northern Henan Province in 2006, the occurrences of *F. bidentis* in this region have continued to increase (Li et al., 2006; Zheng et al., 2018). As of 2021, a large area of *F. bidentis* has been identified in several counties located in the two northernmost cities of Henan Province. The region of these two cities, Anyang City and Hebi City (Figure 2), are the southward expansion frontier zone of *F. bidentis* and are the research focus area of this study. Our aim was to identify the spatial distribution pattern of *F. bidentis* in this expansion frontier zone and to explore the function of frequently distributed habitats (hereafter named preferred habitats) and associated factors that shape the spatial distribution patterns of *F. bidentis*. Since the seeds of *F. bidentis* can be readily dispersed as contaminants of a commodity or by attaching to vehicles, *F. bidentis* has a dispersal advantage with multiple long‐distance vectors (Lu et al., 2009; Zheng et al., 2018), such as trucks, cars, and transporting commodities. Coupled with weak natural dispersal capacity (Horvitz et al., 2017), we speculate that the spatial pattern of *F. bidentis* is discontinuous distribution. We tested this hypothesis by identifying the number of isolated aggregation patches present in recently populated areas. The results of this study could enable more effective prevention and control measures before large areas of *F. bidentis* spread further and cause harm, which is important for protecting the agricultural and ecological security of China.

## 2 MATERIALS AND METHODS

### 2.1 Spatial distribution map visualizing sampling and occurrence points of *Flaveria bidentis*

Historical distribution data of *F. bidentis* in our study area (8595 km^2^), Anyang and Hebi cities, Henan Province, were obtained from various available sources, including herbarium records, published literature (Li et al., 2006; Zheng et al., 2018), and our continuous field investigations between 2006 and 2021. The collected data were then screened and filtered for the following analysis. Only data with complete spatial description information, including occurrence time, spatial location, and habitat, were retained for the spatial distribution pattern analysis. The occurrence data with only spatial location description information were converted to geographic decimal degree coordinates with an online Latitude/Longitude query tool (https://www.toolnb.com/tools/gps.html). In total, 600 sampling locations were investigated, of which 142 locations were found to be populated with *F. bidentis*. Our sampling points covered all of our study areas, with approximately one sampling point per 4 × 4 km grid. Data coordinates were then visualized using ArcGIS 10.2 (ESRI Inc). Data on city and province boundaries were downloaded from the Data Sharing Infrastructure of Earth System Science (http://www.Geodata.cn).

### 2.2 Characterization of the differences in spatial patterns between sampling and occurrence locations of *Flaveria bidentis*

Sampling density can influence the recognition of spatial patterns. When sampling is intensive and systematic, the occurrence points may be considered unbiased. Otherwise, biased sampling data can lead to the misidentification of occurrence patterns. To control for bias, we explored whether or not our distribution pattern of occurrence points could be affected by sampling points. First, we identified the spatial pattern of sampling locations and assessed the spatial pattern differences between sampling and occurrence locations. We employed a nearest neighbor analysis (NNA) to evaluate the distribution types of sampling and occurrence points because of its ability to analyze the spatial patterns of location data by calculating the distance between the nearest points (Wang et al., 2019). The nearest neighbor analysis measures the mean distance of each point to its nearest neighbor and compares the mean distance to what would have been expected in a random distribution of nearest neighbors. This discrepancy of mean distances between the measured distances and the expected distances was expressed with the nearest neighbor index (NNI):

(1) $$\mathrm{NNI}=\frac{r}{\mathrm{Er}}$$

where *NNI* is the nearest neighbor index, *r* is the mean distance of the nearest pair of observed points, and *Er* is the mean distance in the random distribution model. When *NNI* = 1, there is no discrepancy between the expected distances in random distribution and the measured distances in the actual distribution. In contrast, when *NNI* < 1, the points are more clustered than would be expected under a random distribution model. Likewise, when *NNI* > 1, the points are more dispersed than would be expected in a random distribution.

The *r* and Er can be calculated with the following equations:

(2) $$r=\frac{1}{n}\sum_{i=1}^{n}\min\left(\left.d_{\mathrm{ij}}\right|\forall j\right)$$

(3) $$\mathrm{Er}=0.5\sqrt{A/n}$$

where *n* s the number of sampling or occurrence points, *d*_*ij*_ is the distance from point *i* to point *j*, ∀j represents all exhaustive points, $\min\left(d_{\mathrm{ij}}\left|\forall j\right.\right)$ represents the distance from the point *I* to the nearest point, and *A* is the total area of Anyang and Hebi cities.

Nearest neighbor analysis was performed in Crimestat III software (https://www.icpsr.umich.edu/CrimeStat/).

### 2.3 Quantifying the number of occurrence clustering patches (hot spots) of *Flaveria bidentis*

When the occurrence points are clustered distributions, at least one clustering patch should be present. In this study, a single patch was considered a continuous front distribution, while more than one isolated patch was considered a discontinuous front distribution (Mistro et al., 2012; Petrovskaya et al., 2017; Williams & Krock, 2012). To identify the number of clustering patches, we performed a hot spot analysis on the occurrence points of *F. bidentis*. “Hot spot” can be defined as the geographic area representing a small percentage of the study area that contains a high percentage of the studied phenomenon. In our study, a hot spot refers to the patch where the occurrence points were clustered. Nearest neighbor hierarchical clustering (NNH) with CrimeStat III software was employed to identify hot spots. In nearest neighbor hierarchical clustering, points were identified as hot spots if the distances between them were significantly smaller than the mean distance computed under the random distribution model (Wang et al., 2006). This process creates a number of first‐order clusters. Then, it runs the analysis again on the first‐order clusters and circles clusters that are unusually close together to yield second‐order clusters. This iterative process continues establishing more levels of clusters until it can no longer identify any clusters. Therefore, the first‐order clusters are the grouping of points, whereas the higher‐order clusters are groupings of the lower‐order clusters (Levine, 2006; Wang et al., 2006).

During the nearest neighbor hierarchical clustering analysis process, the following parameters were optimized. First, to ensure an objective measure based on probability, we chose the type of search radius, basing the threshold distance on the expectations of a random distribution. We then adjusted the search radius bar to ensure 99.95% confidence that the pairs of points were truly clustered and set the minimum number of points to 2, reducing the likelihood that CrimeStat would identify false positives in its nearest neighbor hierarchical clustering analysis. Furthermore, we output the hierarchical nearest‐neighbor clusters as ellipses and adjusted the size of the ellipses to standard deviations of 1.5 to prevent the ellipse from being too small to be seen and too large to exaggerate the size of the hot spot.

In addition to investigating the number of hot spots, we also examined the shape of each hot spot. Since the occurrence has not occupied the whole range of invaded habitats, the number of hot spots represented the overall distribution pattern of *F. bidentis*, while the shape of each hot spot represented the direction of the spread of *F. bidentis*. Shape parameters of hot spots were estimated by calculating the ratios of the short axis to the long axis. The passible interval of the ratio is (0, 1). A lower ratio indicates a more elongated shape of the clustering patch, while a higher ratio indicates a more rounded shape of the clustering patch. We considered the clustering patch to be an elongated shape if the ratio was less than 0.5, while an approximately rounded shape was assigned if the ratio was greater than 0.5.

The hot spot analysis tool in Crimestat III software was used to generate the clustering patch polygon (hot spot). The number of clustering patch polygons and their shape parameters were calculated using ArcGIS 10.2 software (ESRI Inc).

### 2.4 Screening the preferred habitats of *Flaveria bidentis*

To identify where the clusters were distributed, we screened the preferred habitats of *F. bidentis* in our study area. We first performed a comparative analysis on the sampling and occurrence points habitat types to screen for the frequently measured occurrence habitats of *F. bidentis* by calculating the proportion of different habitat types. We identified the habitat types using land use remote sensing monitoring data at 30 m resolution in 2018. The data were obtained from the Resource and Environment Science and Data Center, Chinese Academy of Sciences (https://www.resdc.cn/). According to the code for classifying urban land use and planning standards of development land in China (https://www.mohurd.gov.cn), the land use in our study area was divided into 13 habitat types (Table 2). We then identified the main habitats of occurrence points in the identified cluster patches. In particular, we also analyzed the habitats of the earliest occurrence in each identified cluster patch.

### 2.5 The function of preferred habitats in the spread of *Flaveria bidentis*

The occurrence preference of a non‐native species in one habitat means that the habitat can provide suitable survival conditions or may can help facilitate its dispersal (Christen & Matlack, 2009). To discern the potential function of the preferred habitat for the persistence and spread of *F. bidentis*, we compared the frequency of sampling and occurrence points with the distances from the habitat. This could describe that when the decay rate of frequency of occurrence points was significantly faster than sampling points, the occurrence points were more concentrated around the habitat than the sampling points. In contrast, this model appears to only provide a suitable habitat when the spatial difference between the sampling points and occurrence points is not significant.

To analyze whether the rate of frequency decay at the occurrence points with increasing distance from the frequently distributed habitat was significantly higher than at the sampling points, we used an exponential decay function to fit the relationship between frequency and distance. The fitted curve was calculated with bootstrap analysis according to the following formula:

(4) $$f\left(x\right)=N_{0}e^{-\mathrm{bx}}$$

where *f(x)* is the frequency at the distance *x*, *N*_*0*_ is the frequency at the first smallest distance (i.e., initial value), and *b* is the decay rate of the frequency as distance from the habitat increases. A higher b‐value implies a faster rate of frequency decay and a more clustered distribution of sampling points or occurrence points around the habitat. We combined the R‐squared and the confidence level, P, to evaluate the goodness of fit. The R‐squared value ranges from 0 to 1, with a value closer to 1 indicating a better fit of the exponential decay curves to the change in frequency. The confidence level, P, described the reliability of the model, and a value closer to 0 indicates a better fit. The confidence level for the exponential decay curves was estimated based on the 1000 re‐sampling iteration bootstrap technique to identify differences between sampling points and occurrence points (Davison & Hinkley, 1997). All calculations were performed using the Python software program.

## 3 RESULTS

### 3.1 Spatial patterns of sampling and occurrence points of *Flaveria bidentis*

Figure 2 visualizes the distribution map of sampling and occurrence points in the study area. Sampling points covered all counties of our study area, while occurrence points were only prevalent in the northern and central counties (Figure 2).

The nearest neighbor analysis showed that the NNI of sampling points was 1.01, which indicated that the actual mean distance between nearest neighbors (2.44 km) was similar to the expected random mean distance between nearest neighbors of sampling points (2.41 km). Therefore, the spatial pattern of sampling points is considered to be randomly distributed. The NNI of occurrence points of *F. bidentis* was 50, which indicated that the actual mean distance between the nearest neighbors (2.04 km) was significantly different from the expected random mean distance between the nearest neighbors of occurrence points (4.09 km) (*p* < .01). Accordingly, the spatial pattern of occurrence points is clustered distribution.

### 3.2 Number of hot spots of clustered occurrence points of *Flaveria bidentis*

**FIGURE 3:**
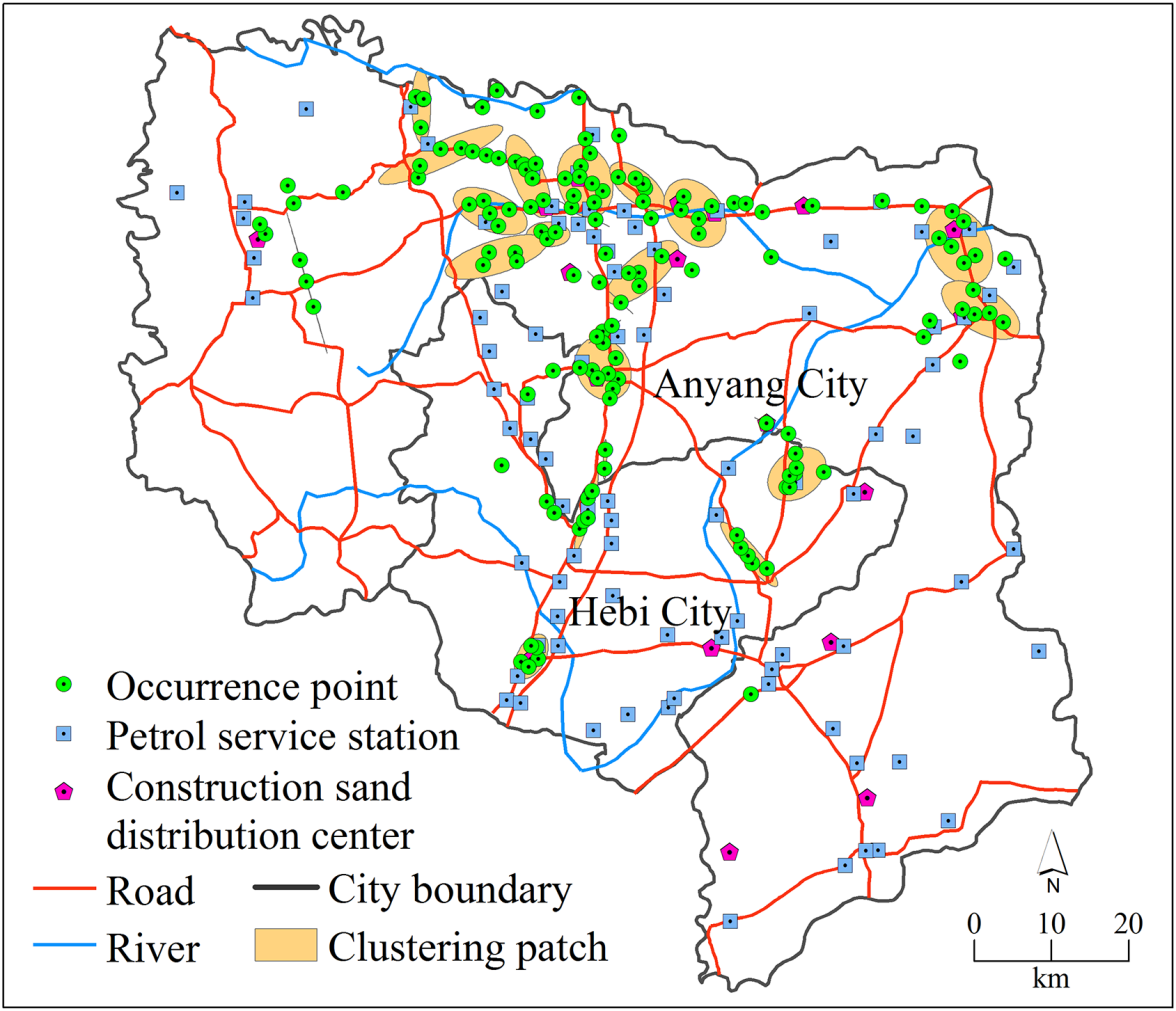
Spatial clustering patches of occurrence points of *Flaveria bidentis* in the study area

Because the spatial distribution pattern of *F. bidentis* was clustered distribution, there could be at least one or more clustered patches. The hot spot analysis described here identified 25 first‐order clusters (orange) and one second‐order cluster (green) in the study area (Figure 3). The 25 first‐order clusters were relatively independent ellipses, mainly concentrated in the northern and central of our study area. The second‐order cluster was located in the north‐central part of our study area. A total of 112 points were included within these clusters, accounting for 79% of all occurrence points (Table 1) at a 99.95% confidence level. The presence of more than one clustering patch suggests that the distribution pattern of *F. bidentis* in recently populated areas is discontinuous.

**TABLE 1:** Ratios of long axis to short axis of clusters

Cluster	Number of points	Length of the long axis (km)	Length of the short axis (km)	Ratio of short axis to long axis
1	9	2.60	2.13	0.819
2	8	2.94	2.21	0.752
3	5	2.99	2.18	0.729
4	5	3.40	2.42	0.712
5	7	2.61	1.83	0.701
6	5	8.44	5.79	0.686
7	5	1.94	1.13	0.582
8	3	1.77	0.98	0.554
9	5	3.55	1.72	0.485
10	5	3.20	1.53	0.478
11	5	2.66	1.24	0.466
12	4	3.62	1.42	0.392
13	8	3.50	1.29	0.369
14	3	4.31	1.31	0.304
15	4	2.99	0.76	0.254
16	5	3.37	0.70	0.208
17	3	5.51	1.02	0.185
18	2	1.02	0.15	0.147
19	5	3.24	0.42	0.130
20	2	1.57	0.15	0.096
21	3	3.29	0.04	0.012
22	2	1.61	0.01	0.006
23	2	2.1	0.01	0.005
24	2	2.31	0.01	0.004
25	2	2.49	0.01	0.004
26	3	5.78	0.02	0.003

The ratios of the short axis to the long axis of clustering patches were 0.003–0.819. Specifically, eighteen clusters had a ratio less than 0.500 (elongated shape) and accounted for 69% of the total clusters. Eight clusters had a ratio greater than 0.500 (rounded shape) and accounted for 31% of the total clusters (Table 1).

### 3.3 Preferred habitats of *Flaveria bidentis*

**FIGURE 4:**
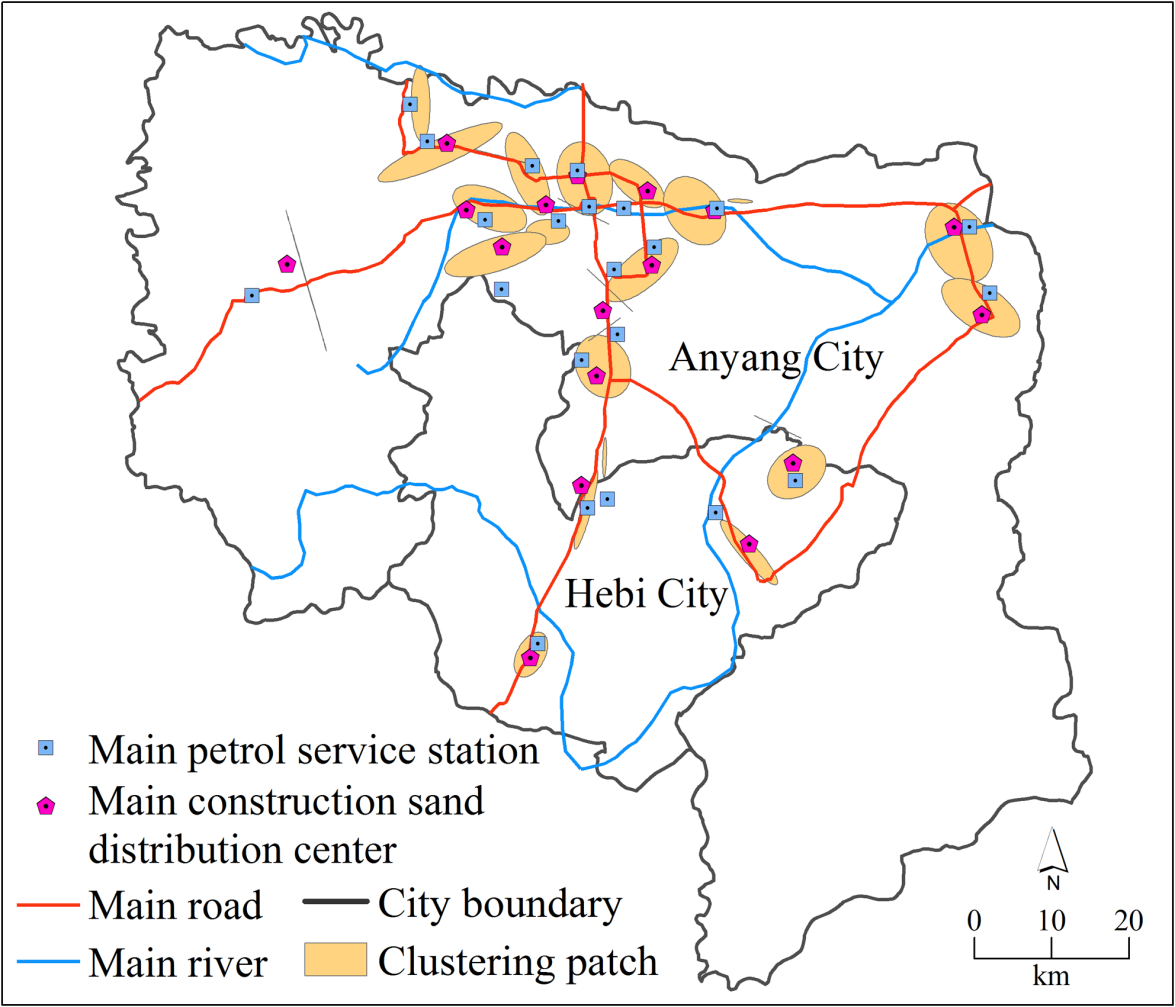
The main roads, petrol service stations, rivers, and construction sand distribution centers where the occurrence clustering patches are located.

There are many factors contributing to the discontinuous distribution pattern of the observed points. Through the investigation, we found that the sampling points (600) were distributed in 13 habitat types while the occurrence points (142) were only present in eight habitat types (Table 2). The number of occurrence points distributed on the roadside, construction sand distribution centers, petrol service stations, and river banks accounted for 83.8% of the total. The clustered occurrence points were only distributed on the roadside (42.2%), construction sand distribution centers (36.7%), petrol service stations (12.6%), and river banks (8.5%) (Figure 4). There were only two habitat types at the earliest occurrence points for each of the hot spots, including roadside (56.4%) and construction sand distribution centers (43.6%).

**TABLE 2:** Habitats of sampling and occurrence points of *Flaveria bidentis*

Habitats	Sampling points		Occurrence points	
	Number	Proportion	Number	Proportion
Farmland	83	13.8%	6	4.2%
Roadside	68	11.3%	52	36.6%
Distribution center of agricultural products	60	10.0%	6	4.2%
Forestry land	55	9.2%	0	0.0%
River bank	52	8.7%	15	10.6%
Petrol service station	48	8.0%	18	12.7%
Construction sand distribution center	46	7.7%	34	23.9%
Grain processing factory	42	7.0%	0	0.0%
Wasteland	41	6.8%	7	4.9%
Construction stone distribution center	39	6.5%	0	0.0%
Nursery garden	32	5.3%	0	0.0%
Park	18	3.0%	1	0.7%
Bus and railway station	16	2.7%	3	2.1%

### 3.4 Function of preferred habitats for the spread of *Flaveria bidentis*

**FIGURE 5:**
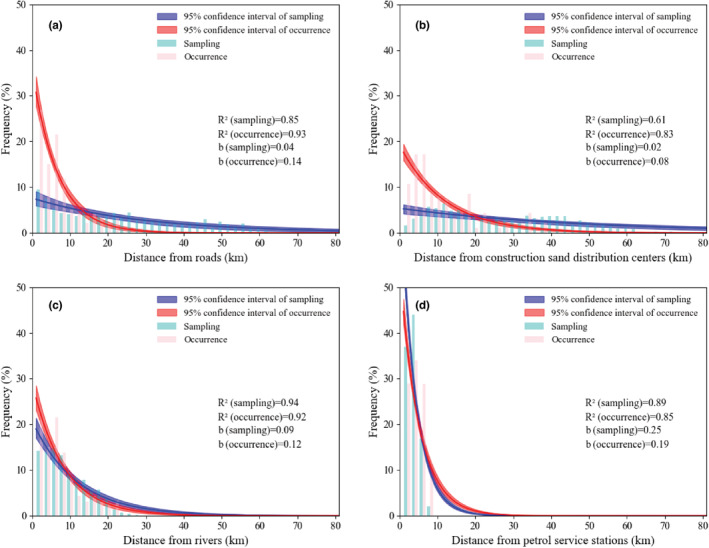
Frequency of sampling points (light blue) and occurrence points (light red) with the increase of the distance from the habitats (a, roads; b, construction sand distribution centers; c, rivers; d, petrol service stations). The lines were fitted with the exponential decay function, and the shaded regions show 95% credible intervals for sampling points (blue) and occurrence points (red).

To further identify the function of preferred habitats, the frequency of sampling and occurrence points from roads, construction sand distribution centers, petrol service stations, and rivers were fitted to an exponential decay function (*R* ^2^ ≥ 0.60, *p* < .01) (Figure 5). However, within the same habitat, there were differences in the goodness of fit between the sampling points and the occurrence points. For example, in roads, the fit accounts for 85% of the total variation in the sampling points around the average, while it accounts for 93% in the occurrence points. Likewise, in construction sand distribution centers, the fit explains 61% of the total variation in the sampling points around the average, while it explains 83% in the occurrence points. Furthermore, in rivers, the fit explains 94% of the total variation in the sampling points around the average and 92% in the occurrence points. Finally, in petrol service stations, the fit explains 89% of the total variation in the sampling points around the average and 85% in the occurrence points.

Similarly, the decay rate (b value) of the frequency of occurrence points from roads and construction sand distribution centers was higher than that of the sampling points, while the decay rates of occurrence and in sampling points from the river and petrol service stations were almost equal (Figure 5). In roads, the decay rates of the frequency of sampling points and occurrence points were 0.04 and 0.14, respectively (Figure 5a). The b value of occurrence points was 4 times greater than the sampling points (Figure 5a). In construction sand distribution centers, the decay rate of the frequency of sampling points and occurrence points were 0.02 and 0.08, respectively (Figure 5b). The b value of occurrence points is 4 times greater than sampling points (Figure 5b). In rivers, the decay rates of the frequency of sampling points and occurrence points were 0.09 and 0.12, respectively (Figure 5c). The b value of occurrence points was 1.33 times greater than sampling points (Figure 5c). In petrol service stations, the decay rates of the frequency of sampling points and occurrence points were 0.25 and 0.19, respectively (Figure 5d). Furthermore, the b value of occurrence points is 0.76 times greater than sampling points (Figure 5d).

## 4 DISCUSSION

### 4.1 The patchy distribution pattern of *Flaveria bidentis* on the edge of its expanding range in China

Understanding the spatial patterns of a non‐native species in newly introduced areas is a problem of considerable theoretical and practical importance (Burkart, 2001; Petrovskaya et al., 2017; Shigesada & Kawasaki, 2010). Much of the literature has focused on the distribution patterns of non‐native species and distinguishing between continuous and discontinuous spatial distribution patterns of new populations of new non‐native species (Mistro et al., 2012; Rodrigues et al., 2015). Kolmogorov et al. (1937) and Skellam (1951) suggested that the existence of a continuous front separating a recently populated area behind from a non‐native free area is a general characteristic of invasive spread. This view was eventually challenged by the discovery that there are alternate scenarios of “patchy invasion” in which no continuous front exists and the non‐native species occurs on separate patches with a high density of *F. bidentis* (Petrovskii et al., 2005; Rodrigues et al., 2015). In recent decades, discontinuous distribution has become the more prevalent spatial distribution pattern of non‐native species at the dispersal front zone (Mistro et al., 2012; Morozov et al., 2006; Petrovskii et al., 2002).

The results of our analysis of the spatial distribution pattern confirmed that *F. bidentis* presents discontinuous spatial patterns in newly populated areas. We found the difference between the spatial patterns of sampling points and actual occurrence points, with sampling points being random while occurrence points were clustered. The random distribution characteristics of sampling points in our survey data over 15 years were objective and comprehensive. However, the cluster distribution characteristics of occurrence points indicated that there might be one or more spatially clustered patches of *F. bidentis*.

The result of hot spot analysis found that there were 25 independent spatial clustering patches of occurrence points. Multiple clustering patches indicated that the occurrence points of *F. bidentis* presented discontinuous spatial patterns. This discontinuous distribution pattern is heterogeneous because the clustering patches were heavily populated, and the others nearby are sparsely populated or not populated at all. Further analysis of the shape of these clustering patches found that the ratios of the short axis to the long axis were 0.003–0.819, in which 69% of the ratios were less than 0.5. This means that these clustering patches exhibited an elongated shape. In theory, when non‐native species populate a new zone, the ability to spread towards the periphery relies on its own dispersal capacity to be constant. However, the elongated shape of the clustering patches indicates that the spread rate of *F. bidentis* is anisotropic, with faster dispersal in the direction of the long axis and slower dispersal in the direction of the short axis. Most of the aggregation areas had a long axis to road axis of less than 45°, which indicated that the extension direction of the *F. bidentis* population was more consistent with the direction of the road.

The differences between the spatial distribution patterns of sampling points and actual occurrence points, coupled with the spatial heterogeneity at different levels of both the number and shape of the cluster, indicated that there could be many factors contributing to the appearance of the clustered distribution of *F. bidentis*. Several scholars have reported that the climate of our study area was suitable for *F. bidentis*, implying that it is capable of continuously spreading to surrounding areas (Bai et al., 2009; Zheng et al., 2018). However, the occurrence points show a discontinuous distribution pattern, and there were several independent clustering patches. Moreover, the clustering patches and earliest occurrences were located in the human disturbance habitats, including roadsides and construction sand distribution centers. Therefore, we concluded that the patchy spatial pattern is generated by additional influencing factors such as human‐mediated factors that dominate the long‐distance dispersal of its seeds or a vegetative propagule.

### 4.2 Unintentional seed movement as commodity contaminant are dominant factors underlying patchy distribution patterns

Our results on preferred habitats of *F. bidentis* showed that the occurrence points are frequently distributed at roadsides, construction sand distribution centers, petrol service stations, and river banks. Previous studies have demonstrated that *F. bidentis* has a strong tolerance to stress and can tolerate salt and alkali, drought, barren, and cold conditions (Fan et al., 2018; Li et al., 2006; Lu et al., 2009; Yan et al., 2016). This successful non‐native can thus survive in a wide range of our sampling habitat types (Table 2). However, the occurrences of *F. bidentis* were mainly distributed in four human‐disturbed habitats. The discontinuous distribution pattern of *F. bidentis* in recently populated areas could be correlated with human activities. Non‐native plants have limited capacity to spread on their own, but their propagules could be unintentionally transferred through trade as a transport‐contaminant of an import commodity or as transport‐stowaway attached to transporting vessels (Hulme et al., 2008). Historically, globalization, along with imported grain, horticultural flowers, and animal trading activities, was the most common introduction route for non‐native species (Hulme, 2009; Rautureau et al., 2011; Wilson et al., 2009). In addition, non‐native species may also undergo dispersal with various transport activities, such as localized stone and sand transport. In our field survey, we found that *F. bidentis* was only distributed around the construction sand distribution centers but not at grain processing factories, nursery gardens, or construction stone distribution centers. This distribution characteristic illustrates that the pattern of spread of *F. bidentis* may be distinct from common activities but is rarely reported that way. The clustering occurrence points were most frequently distributed in the roadsides and construction sand distribution centers, followed by petrol service stations and river banks. Compared with all of the occurrence points, the clustering points have a higher percentage of distribution on roadsides and construction sand distribution centers. We also found that the earliest occurrence points of each clustering patch were mainly distributed around roadsides and construction sand distribution centers, indicating that the human operational activities associated with roads and prefabrication factories could prompt *F. bidentis* to spread between multiple patches.

Further spatial analysis revealed that both sampling and occurrence points have a tendency to decay as the distance from roads, construction sand distribution centers, petrol service stations, and river banks increases. However, in contrast, the goodness of fit and the decay rates of occurrence points are higher in roads and construction sand distribution centers compared with the sampling points. In addition, compared with sampling points, occurrence points were significantly clustered around roads and construction sand distribution centers. Such an occurrence preference of *F. bidentis* means that roads and construction sand distribution centers appear to function as both habitats and movement corridors for *F. bidentis* distribution, whereas rivers and petrol service stations with nonsignificance of the spatial difference between sampling and occurrence points simply provide habitats for *F. bidentis*. All the earliest occurrences found in the two preferred habitats further indicated that roads and construction sand distribution centers functioned as movement corridors and the source of the secondary local spread. The dispersal of species along road corridors has received the attention of many scholars. For example, Christen and Matlack (2009) analyzed the distribution patterns of three non‐native species along the road, highlighting that the roads function not just as a habitat for the non‐native species but also as a conduit for their dispersal. Zheng et al. (2018) confirmed that the roads were the corridor for *F. bidentis* dispersal by analyzing the relationship between distribution points and major roads. Our study indicates that in addition to roads, construction sand distribution centers are also a dispersal conduit for *F. bidentis*. The non‐native plant *F. bidentis* propagates as seeds, which are slight and 2–2.5 mm in length (Li et al., 2006; Tang et al., 2010). The tiny size of the seed allows dispersal as a contaminant in construction or agricultural goods or as a hitchhiker adhered to wheels and thus transported by vehicles over long distances. Therefore, human activities associated with major roads and construction sand distribution centers could be affecting the its dispersal.

Activities related to the sand trade for construction are the link between roads and construction sand distribution centers as dispersal mechanisms. Sand for construction is usually extracted in a sand mining field, then transported to the construction sand distribution centers in vehicles, and finally transferred to areas where sand is used. Considering our field investigation and spatial analysis results, we hypothesize that the trade of construction sand could be the dominant driving factor for *F. bidentis* dispersal and introduction to new areas. Mechanistically, the slight and tiny seeds of *F. bidentis* could adhere to sand transported by wheels and fall on roads during transport. Later, they are moved by wind to wasteland and greening bands on either side of the road and begin to grow, thus exhibiting a spatial distribution pattern that extends along both sides of the road (Zheng et al., 2018). Additionally, the seeds of *F. bidentis* contaminated in the sand could be transported to a transfer station or sandy land along with the sand over long distances. Due to a lack of management during sand loading, *F. bidentis* seeds spatially exhibit discrete and separate distribution zones after escaping to form populations in suitable habitats near these sites.

### 4.3 Implications for optimizing management strategies to prevent further spread

Once persistent in an area, non‐native species can spread further. Successfully persistent populations could be the source of secondary spread, leading to a self‐accelerating process whereby spread in a new area results in spread in a new area (Bertelsmeier & Keller, 2018). For example, in our study, multiple independent clusters could all be the source of the secondary spread of *F. bidentis*. Therefore, to effectively inhibit the spread of this plant, it is important to take prevention and control measures for all of the clusters. Our results identified the spatial distribution of *F. bidentis* clusters, and found that the factors driving spread were roads and construction sand distribution centers. Therefore, we suggest that removal measures be taken on the interior of clusters to reduce the possibility of secondary spread from an already populated cluster. Since *F. bidentis* already forms a denser distribution inside clusters, these areas need physical or chemical controls, such as removal with machinery or spraying pesticides. Similarly, to control the spread of *F. bidentis* along movement corridors, focused control should be performed on roads and construction sand distribution centers. If *F. bidentis* is in these movement corridors, we may be able to completely eradicate it by the roots in a timely manner. Furthermore, since roads and construction sand distribution centers are major sites of human activity, and *F. bidentis* seeds are prone to attach to goods, we recommend monitoring the logistics of activities related to both. Similarly, monitoring should also be undertaken in hub areas where these stream activities operate.

## 5 CONCLUSION

Understanding distribution patterns and their driving factors in newly populated areas is key to the early detection of non‐native species. However, few studies properly and rigorously address the mechanisms contributing to the distribution patterns of non‐native species with adequate sampling, largely due to financial and labor constraints (Horvitz et al., 2017; Wang, Wang, et al., 2011). Based on field investigation data from nearly 15 years from the newly populated areas, our research combines field distribution data with a spatial analysis model to identify the discontinuous distribution pattern of *F. bidentis* in newly populated areas and further explores the driving factors behind its distribution pattern. Critically, this study links major roads and construction sand distribution centers as the major driving factors contributing to the spatially heterogeneous distribution pattern of *F. bidentis* in its expansion frontier zone. This ecological disturbance caused by human activity along sand trade trajectories has facilitated the growth and dispersal of populations of non‐native species. This understanding is crucial for developing appropriate and efficient strategies for preventing and mitigating the introduction and persistence of non‐native species.

## AUTHOR CONTRIBUTIONS

**Qianmei Wu:** Conceptualization (equal); data curation (lead); formal analysis (lead); investigation (equal); methodology (equal); writing – original draft (lead); writing – review and editing (equal). **Chengdong Xu:** Methodology (supporting); software (supporting); writing – review and editing (supporting). **Jiamei Li:** Investigation (supporting); methodology (supporting); writing – review and editing (supporting). **Wan Xue Liu:** Methodology (supporting); writing – review and editing (supporting). **Fanghao Wan:** Methodology (supporting); writing – review and editing (supporting). **Jianying Guo:** Conceptualization (equal); data curation (supporting); writing – review and editing (supporting). **Rui Wang:** Conceptualization (lead); formal analysis (equal); investigation (equal); methodology (equal); writing – review and editing (equal).

## CONFLICT OF INTEREST

The authors have no conflict of interest to declare.

## Data Availability

The data that support the findings of this study have been archived through Dryad online data repository and the URL was https://datadryad.org/stash/share/j3DnvLdg5MZrg4‐4SEZYscmLsWBdFl9c8P5Hha9qMjo.
